# Curcumin loaded sub-30 nm targeting therapeutic lipid nanoparticles for synergistically blocking nasopharyngeal cancer growth and metastasis

**DOI:** 10.1186/s12951-021-00966-6

**Published:** 2021-07-28

**Authors:** Haiming Luo, Lisen Lu, Ni Liu, Qingqing Li, Xiaoquan Yang, Zhihong Zhang

**Affiliations:** 1 Britton Chance Center for Biomedical Photonics, Wuhan National Laboratory for Optoelectronics-Huazhong University of Science and Technology, Wuhan, 430074 China; 2 MoE Key Laboratory for Biomedical Photonics, Department of Biomedical Engineering, Huazhong University of Science and Technology, Wuhan, 430074 China

**Keywords:** Nasopharyngeal carcinoma, Targeted therapy, Curcumin, Peptide, Lipid nanoparticles

## Abstract

Systemic chemotherapy is still the primary treatment for advanced-stage nasopharyngeal carcinoma (NPC), but only limited therapeutic success has been achieved in the past decade because of drug resistance and systemic toxicity. Curcumin (Cur) is an effective alternative to chemotherapeutics because it showed remarkable therapeutic potential in the treatment of NPC. However, lack of tissue specificity and poor penetration in solid tumors are the major obstacles to effective therapy. Therefore, in this work, a self-assembled sub-30 nm therapeutic lipid nanoparticle loaded with Cur, named as Cur@α-NTP-LN, was constructed, specifically targeting scavenger receptor class B member 1 (SR-B1) and enhancing its therapeutic effects on NPC in vivo. Our results showed that Cur@α-NTP-LNs were effective and superior to free Cur on NPC cell-specific targeting, suppressing cell proliferation and inducing cell apoptosis. In vivo and ex vivo optical imaging revealed that Cur@α-NTP-LNs exerted high targeting efficiency, specifically accumulating in NPC xenograft tumors and delivering Cur into the tumor center after systemic administration. Furthermore, Cur@α-NTP-LNs exhibited a remarkable inhibitory effect on the growth of NPC subcutaneous tumors, with over 71 and 47% inhibition compared to Cur- and α-NTP-LNs-treated groups, respectively. In addition, Cur@α-NTP-LNs almost blocked NPC metastasis in a lung metastasis model of NPC and significantly improved the survival rate. Thus, the sub-30 nm Cur@α-NTP-LNs enhanced the solubility of Cur and demonstrated the ability of targeted Cur delivery into the center of the solid NPC tumor, performing synergistic inhibitory effects on the growth of NPC tumor and its metastasis with high efficiency.

**Supplementary Information:**

The online version contains supplementary material available at 10.1186/s12951-021-00966-6.

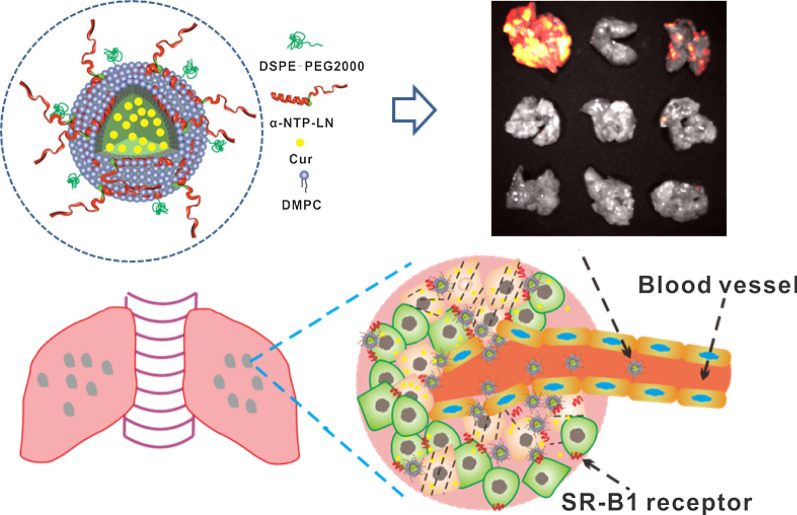

## Introduction

Nasopharyngeal carcinoma (NPC) is a malignant head and neck cancer with high incidence in Southern China and Southeast Asia [1, 2]. NPC is characterized by an aggressive feature, poor diagnosis, and limited therapeutic opportunities [2, 3]. The outcome of metastatic NPC is still disappointing, and failure on distant metastasis remains a significant challenge. Palliative chemotherapy plays a significant role in advanced or metastatic NPC and could potentially prolong the survival of patients with advanced NPC [3]. Current standard treatments for advanced NPC involve platinum-based doublet chemotherapy consisting of cisplatin/carboplatin plus gemcitabine, paclitaxel, or 5-FU, which act on cell death effectors to induce cell apoptosis and inhibit cell survival of cancer cells [4]. Although these chemotherapeutic drugs have the advantages of rapid action and ready availability, the overall survival of NPC patients is far from satisfactory and side effects are intolerable to most patients [5]. Targeted therapies on epidermal growth factor receptor (EGFR) and vascular endothelial growth factor receptor (VEGFR) have been tested in NPC patients in clinical practice according to the molecular biological characteristics of NPC [6]. Targeting a single pathway usually fails to achieve an adequate diagnosis and effective treatment due to the inherent complexity and heterogeneity of cancer [7, 8]. Unlike other chemotherapeutic agents, Curcumin (Cur) is well tolerated and exhibits specific anti-cancer, antioxidant, and anti-inflammatory effects, which are mediated through the regulation of numerous targets such as nuclear factor (NF-κB), transcription factor activator protein-1 (AP-1), and tumor-associated growth factors [9]. Many preclinical studies demonstrated that Cur can be more effective than a single pathway-targeted anti-cancer drug thanks to its pleiotropic properties [10]. Unfortunately, the poor pharmacokinetics and poor bioavailability of Cur in vivo hinder its clinical translation.

Drug delivery systems are designed to provide localized or targeted delivery of chemotherapeutic agents, resulting in an efficient approach for improving the solubility and bioavailability of Cur [11–13]. Various drug delivery carriers such as liposomes, polymeric micelles, and lipid nanoparticles have been reported to successfully encapsulate Cur to increase its solubility, thus enhancing its bioavailability and therapeutic effects [14, 15]. These Cur nanoformulations have different characteristics, tailored by modulating the mechanisms of inducing cell death [16–18]. Although they offer various advantages for effective chemotherapy, lack of tissue and cell specificity is still a significant obstacle [14]. Furthermore, most of these conventional nanoparticles are generally over 50 nm in size after drug encapsulation, thus, the penetration into solid tumor tissues (e.g. pancreatic cancer and NPC) is difficult, making difficult the achievement of satisfactory treatment effects [19]. In our previous report, a hybrid peptide was designed by the fusion of an amphipathic α-helical peptide with an NPC-specific therapeutic peptide, to interact with phospholipids, and it was self-assembled into sub-30-nm lipid nanoparticles (named as α-NTP-LNs) that exhibited promising therapeutic effects on NPC tumors and metastases [20]. In this work, Cur was encapsulated into α-NTP-LNs to achieve a better-targeted therapeutic modality, and the newly formed Cur@α-NTP-LNs could precisely deliver Cur to tumor sites with enhanced treatment efficacy.

Therefore, the aim of this work was to develop a therapeutic nanoparticle formulation combining the specific NPC targeting ability of α-NTP-LNs and anticancer effects of Cur, to form an attractive agent for delaying NPC cancer onset and progression. Cur was encapsulated into the lipid nanoparticles α-NTP-LNs, and the final preparation Cur@α-NTP-LNs was optimized to ensure its stability and NPC-specific targeting ability. Importantly, the targeted delivery properties of Cur by Cur@α-NTP-LNs, in vivo pharmacokinetics and therapeutic effects of Cur@α-NTP-LNs on the growth of NPC subcutaneous tumor and its metastasis were evaluated.

## Materials and methods

### Materials

1,2-Dimyristoyl-sn-glycero-3-phosphocholine (DMPC) was purchased from Avanti Polar Lipids Inc. (Alabaster, Alabama, USA). Hoechst 33,342, free Cur, and DSPE-PEG2000 were purchased from Sigma-Aldrich Co. (St. Louis, MO, USA). DiR-BOA was synthesized according to our procedure previously described [21, 22]. Free α-NTP with the sequence FAEKFKEAVKDYFAKFWDGSGLTVSPWYLTVSPWY was purchased from Shanghai Apeptide Co., Ltd. (Shanghai, China). APC Annexin V Apoptosis Detection Kit with propidium iodide (PI) was purchased from Biolegend (Pharmingen, San Diego, CA, USA).

### Preparation of Cur@α-NTP-LNs and Cur(DiR-BOA)@α-NTP-LNs

A similar procedure used to synthesize α-NTP-LNs previously described was used to prepare Cur@α-NTP-LNs [20]. The mixture of DMPC (3 μM), DSPE-PEG2000 (0.7 μM), and Cur (0.7 μM) in chloroform was dried under nitrogen to form a uniform lipid film. After adding 1 ml phosphate-buffered saline (PBS), the mixture was sonicated at 48°C for 1 h to form a lipid emulsion. PBS containing different amounts of α-NTP peptide was added into the lipid emulsion to make Cur@α-NTP-LNs by an overnight incubation at 4°C. As regard the preparation of the nanoparticles Cur(DiR-BOA)@α-NTP-LNs, which were core loaded with DiR-BOA, the only difference in the above-described protocol was the first step, in which 0.2 μM of DiR-BOA was mixed with DMPC, DSPE-PEG2000, and Cur in chloroform. The nanoparticles were concentrated using a concentrator tube with a 30-kDa molecular weight cutoff (Millipore, USA). The quantification of the proteins in the nanoparticles was evaluated using an mp06667-CBQCA protein quantitation kit (Invitrogen Corporation, California, CA, USA) and Pierce Modified Lowry Protein Assay Kit (Thermo Fisher Scientific; Rockford, IL, USA).

### Morphology, size, and spectrum of the NPs

The morphology and size of Cur@α-NTP-LNs were evaluated using a Tecnai G^2^ 20 U-Twin transmission electron microscope (FEI Company, USA). The particle size distribution and zeta potential were measured using a dynamic light scattering (photon correlation spectroscopy) on a Zetasizer Nano-ZS90 system (Malvern Instruments, Worcestershire, UK). The spectrum of free Cur and Cur@α-NTP-LNs was measured with a spectrophotometer (Lambda 35; PerkinElmer, Waltham MA, USA).

### Stability evaluation

Cur(DiR-BOA)@α-NTP-LNs were run on a 8% semi-native SDS–polyacrylamide gel electrophoresis (SDS-PAGE) to ensure whether the fluorescent band of free Cur matched with DiR-BOA with a custom-made optical fluorescence imaging system [23, 24]. The nanoparticles were incubated with PBS, 10% FBS, 10% plasma, and 5% Tween-20 at 37°C for 3 h before running the gels.

### In vitro drug release by Cur@α-NTP-LNs

To measure the release kinetics of Cur by Cur@α-NTP-LNs, 8 aliquots of 200 μL Cur@α-NTP-LNs solution were placed into 8 tubes. After incubation for 0, 3, 6, 12, 15, 24, 48, and 72 h at 37°C, each tube was centrifuged using a table-top centrifuge (ATT-101, HITECH Co., Ltd., Tokyo, Japan) at 10,000 g for 2 min. The supernatants were transferred to 96-well plates and measured with a fluorometric imaging plate reader (Flexstation 3; Molecular Devices Corp., Sunnyvale, CA, USA).

### Cell culture

Human nasopharyngeal cancer cells 5-8F were donated by Prof. Mu-Sheng Zeng (Sun Yat-sen University Cancer Center, Guangzhou, China). mRFP-5-8F cells stably expressing the monomer red fluorescent protein mRFP were screened for whole-body fluorescence imaging of tumor models [25]. 5-8F and mRFP-5-8F cells were cultured in RPMI-1640 (Invitrogen Life Technologies, Carlsbad, USA) supplemented with 10% fetal bovine serum (FBS, Life Technologies), 100 U/ml penicillin, and 0.1 mg/ml streptomycin, and incubated at 37°C under 5% CO_2_.

### Confocal imaging

1 × 10^4^ 5-8F cells were seeded into each well of an 8-well cover glass-bottom chamber (Thermo Scientific, Holtsville, NY, USA) for confocal imaging. Cur@α-NTP-LNs (Cur concentration, 2.5 μM) were incubated with the cells at 37°C for 3 h. After staining with Hoechst 33,342 for 5 min, cells were washed twice with PBS and the fluorescence signals were observed using a laser confocal scanning microscope (LSM710, Carl Zeiss), at an excitation wavelength of 405 nm for Hoechst 33,342 and 488 nm for Cur.

### Cell proliferation and cell killing detection

5-8F cells were seeded into 48-well plates (2 × 10^4^/well) for flow cytometry analysis. Cur@α-NTP-LNs at the concentration of 10, 25, 50, 75, and 100 μM (Cur concentration) were separately incubated with cells at 37°C for 3 h. The fluorescence intensity of cells was examined using a microcapillary flow cytometer (Guava EasyCyte8HT, EMD Millipore Corporation, Billerica, MA, USA) at an excitation wavelength of 488 nm for Cur (525/30 emission filter).

5-8F cells were seeded into 24-well plates at a density of approximately 5 × 10^4^ cells per well. After 24 h incubation with Cur@α-NTP-LNs and Cur at various Cur concentrations (10, 25, 50, 75, 100, 150, and 200 μΜ) and α-NTP-LNs at various peptide concentrations (20, 50, 100, 150, 200, 350, and 400 μΜ), the cells were imaged using a microscope (IX71, Olympus) and the cell viability was assessed by the methyl tetrazolium salt (MTS) assay (Cell Titer 96TM Aqueous; Promega, Madison, WI, USA).

The toxicity of the nanoparticles was evaluated as follows. 5-8F cells were incubated with Cur@α-NTP-LNs and Cur at various Cur concentrations (25, 50, 100, 150, 200, and 250 μΜ) or Cur@α-NTP-LNs and α-NTP-LNs at various peptide concentrations (50, 100, 200, 300, 400, and 500 μΜ) for 24 h, and stained with PI for flow cytometry experiments.

As regard cell apoptosis detection, 5-8F cells were incubated with 100 µM free Cur, Cur@α-NTP-LNs (100 μΜ Cur, 200 μΜ peptides) or peptide equivalent α-NTP-LNs (200 μΜ peptides) for 24 h and then stained with APC-Annexin V and PI for flow cytometry analysis.

### Cell wound healing assay and clonal inhibition assay

5-8F cells were seeded into 6-well plates (5 × 10^5^/well) for 24 h. The cells were scratched with a pipette tip and treated with 50 μM free Cur, Cur equivalent Cur@α-NTP-LNs (50 μΜ Cur, 100 μΜ peptides), or peptide equivalent α-NTP-LNs (100 μΜ peptides). After 24 h, cell migration was evaluated by microscopy (IX71, Olympus) equipped with a 4 × objective.

As regard the colony formation assay, 6-well plates were coated with 2 mL 0.6% soft agar in RPMI 1640 medium supplemented with 10% FBS. 5-8F cells were seeded into plates with 2 mL 0.3% soft agar in RPMI 1640 medium supplemented with 10% FBS and 25 μΜ free Cur, Cur equivalent Cur@α-NTP-LNs (25 μΜ Cur, 50 μΜ peptides) and peptide equivalent α-NTP-LNs (50 μΜ peptides) and incubated for 14 days at 37 °C under 5% CO_2_. The culture medium was changed every two days. The colonies were imaged by microscopy (IX71, Olympus) equipped with a 4 × objective, and their diameters above 50 pixels were considered positive.

### In vivo and ex vivo fluorescence imaging

All animal studies were performed in compliance with the protocols approved by the Hubei Provincial Animal Care and Use Committee and with the practical guidelines of the Animal Experimentation Ethics Committee of the Huazhong University of Science and Technology. A total of 2 × 10^6^ 5-8F cells were subcutaneously implanted into the rear leg of the mice. When tumors reached a diameter of 5–8 mm (2 to 3 weeks after implantation), 200 nmol Cur(DiR-BOA)@α-NTP-LNs or (DiR-BOA)@α-NTP-LNs were intravenously injected into tumor-bearing mice. In vivo and ex vivo fluorescence imaging of the mice and tissues was performed using a custom-made whole-body optical imaging system [23, 24]. The fluorescence signals of DiR-BOA were acquired using a near-infrared filter set (excitation: 716/40 nm; emission: 775/46 nm) and calibrated with an autofluorescence background filter set (excitation: 562/40 nm; emission: 775/46 nm). The fluorescence images of mRFP were acquired using a red filter set (excitation: 469/35 nm; emission: 562/40 nm) and calibrated with an autofluorescence background filter set (excitation: 469/35 nm; emission: 655/40 nm).

### Fluorescence analysis of frozen slices

5-8F tumor-bearing mice were sacrificed by cervical dislocation after the last imaging and the tumors were collected and frozen. The tumor was cut into 10 μm-thick sections using a Shandon FSE cryotome (Leica CM1900, Germany), and the slices were stained by DAPI and imaged using spinning disk confocal microscopy (SDCM, PerkinElmer) using 488 nm excitation and 640 nm emission wavelength and 10 × objective. All data were processed using Matlab and Image J.

### Blood clearance kinetics

Five normal nude mice were used as one group to test blood clearance of Cur(DiR-BOA)@α-NTP-LNs and (DiR-BOA)@α-NTP-LNs. Blood samples were collected from the orbital sinus at different time points post-injection (10 min to 48 h) of the nanoparticles. The fluorescence intensity of DiR-BOA in the blood samples was detected using a spectrophotometer (Lambda 35; PerkinElmer, Waltham MA, USA) and recorded per milligram of blood.

### Inhibitory effect of Cur@α-NTP-LNs on tumor growth

5-8F subcutaneous tumor-bearing mice were divided into four groups on day 13 after tumor cell implantation. Each group contained five mice, and the mice were treated with an intravenous injection of PBS and 125 nmol free Cur, Cur equivalent Cur@α-NTP-LNs (125 nmol Cur, 250 nmol peptides), or peptide equivalent α-NTP-LNs (250 nmol peptides) on day 13, 15, 17, 19, and 21, respectively. Tumor size was measured with a caliper, and the volume was calculated according to the following formula: V = (π/6 × longest diameter × perpendicular diameter^2^).

### Histopathological analyses

Lung metastatic tumors were collected and fixed in a 4% paraformaldehyde solution. The tumors were embedded in paraffin, sectioned, and stained with hematoxylin and eosin (H&E). The images of the H&E staining were acquired using a Nikon A1 laser scanning confocal microscope.

### Hemanalysis and biochemical analyses

Blood samples were collected from 5-8F tumor-bearing mice treated with PBS or 125 nmol free Cur, Cur equivalent Cur@α-NTP-LNs (125 nmol Cur, 250 nmol peptides), or peptide equivalent α-NTP-LNs (250 nmol peptides) on days 13, 15, 17, 19, and 21. The blood cell and biochemical analyses were performed using a hematology analyzer (BC-3200, Mindray, Shenzhen, China) and an automatic biochemical analyzer (Spotchem EZ SP-4430, Arkray Inc., Kyoto, Japan), respectively.

### Effect of Cur@α-NTP-LNs on NPC lung metastasis and survival rate

5-8F cells were intravenously injected into athymic nude mice (nu/nu) and randomly divided into four groups, which were treated with an intravenous injection of PBS or 125 nmol free Cur, Cur equivalent Cur@α-NTP-LNs (125 nmol Cur, 250 nmol peptides), or peptide equivalent α-NTP-LNs (250 nmol peptides). The treatments were started on days 13, 15, 17, 19 and 21 from the injection of the 5-8F cells. Three mice in each group were used to perform an X-ray computed tomography scan on day 25 to detect whether pulmonary metastases occurred in mice with different treatments. After the CT scan, the mice were sacrificed, and lung tissues were collected and subjected to H&E staining. The survival rate was monitored in the remaining 5 mice.

### Statistical analysis

Statistical analysis was performed using GraphPad Prism with log-rank test. Nonparametric statistical tests (Kruskal–Wallis and Mann–Whitney U tests) were used to analyze the significance of the differences in the hemanalysis and biochemical parameters, and two-tailed Student’s *t*-tests was used to analyze the other data. All data are expressed as mean ± standard deviation (s.d.). Differences between or among groups were labeled as n.s. for not significant values, or * for *p* < 0.05, ** for *p* < 0.01, and *** for *p* < 0.001.

## Results

### Characterization and drug encapsulation

**Fig. 1:**
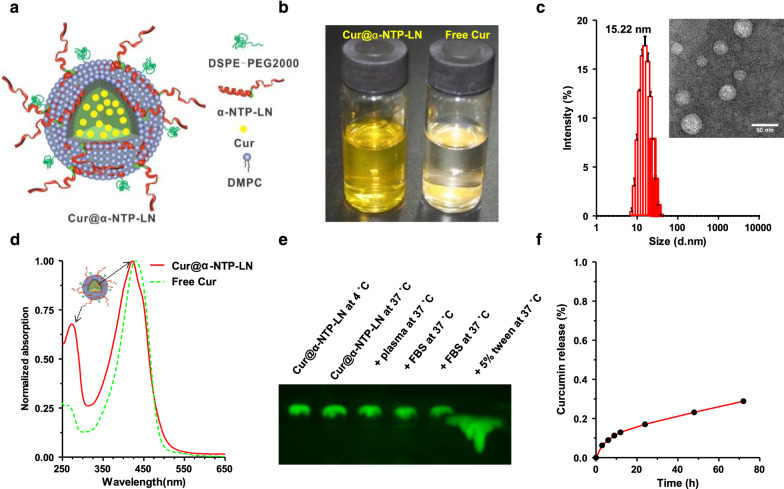
In vitro characterization of Cur@α-NTP-LNs. **a** Schematic diagram of Cur@α-NTP-LNs. **b** Appearance of Cur@α-NTP-LNs (left) and free Cur (right) in PBS. **c** Particle size distribution and typical TEM image of Cur@α-NTP-LNs. **d** UV–visible absorption spectrum of Cur@α-NTP-LNs and free Cur in PBS solution at room temperature. **e** Semi-native SDS-PAGE assay and fluorescence imaging to evaluate the stability of Cur@α-NTP-LNs. **f** Release profile of Cur from Cur@α-NTP-LNs

Different amounts of α-NTP peptide (0.36 to 1.08 μmol) were added to DMPC lipid mixture (3 μmol), Cur (0.7 μmol) and DSPE-PEG2000 (0.7 μM) to optimize the formulation ratio for the fabrication of Cur@α-NTP-LNs. The schematic diagram of Cur@α-NTP-LNs is shown in Fig. 1a. When the turbid emulsion became transparent (Fig. 1b), dynamic light scattering (DLS) measurements were immediately performed. The results showed that the intensity weighted particle size of Cur@α-NTP-LNs varied from 36.1 to 15.2 nm as the amount of α-NTP increased from 0.36 to 0.72 μmol (Additional file 1: Fig. S1). When the amount of α-NTP was increased to 1.08 μmol, the solution became cloudy, and the size of the nanoparticle was 119.8 nm, probably because of the excessive free Cur at high peptide/lipid molar ratio that became insoluble, leading to a lower loading capacity and cloudy emulsion. Thus, 0.72 μmol α-NTP was used to synthesize Cur@α-NTP-LNs due to the ideal size of the nanoparticles and the best loading efficiency of Cur. Transmission electron microscopy (TEM) and dynamic light scattering (DLS) measurements revealed the uniform spherical form of the Cur@α-NTP-LNs with a diameter of 19.8 ± 4.2 nm (Fig. 1c). The absorption spectrum of Cur@α-NTP-LNs confirmed that Cur was successfully loaded into the nanoparticles (Fig. 1d).

Subsequently, Cur@α-NTP-LNs were mixed with an equivalent volume of PBS, 10% FBS or mouse plasma to measure the stability of Cur@α-NTP-LNs, and incubated at 4 or 37ºC for 3 h to run semi-native SDS-PAGE gels. The amount of Cur@α-NTP-LNs in different treatments could be evaluated by directly detecting the fluorescence intensity of Cur in the gel once separated by electrophoresis due to the unique optical properties of Cur (Additional file 1: Fig. S2). Similar fluorescence intensity of Cur in control and treated samples confirmed that Cur@α-NTP-LNs were stable during 3 h incubation in 10% FBS and 10% plasma at 37ºC (Fig. 1e). As a control, the fluorescence imaging of Cur@α-NTP-LNs after dissociation with 5% Triton X-100 on the gel showed diffuse fluorescent signals of Cur (Fig. 1e). The in vitro release profile of the loaded Cur from Cur@α-NTP-LNs at physiological pH is shown in Fig. 1f. Cur was released in a sustained manner, and only 30% of the total Cur was released from Cur@α-NTP-LNs at 80 h of incubation.

### Targeted delivery of Cur@α-NTP-LNs and evaluation of its anti-cancer effects

**Fig. 2:**
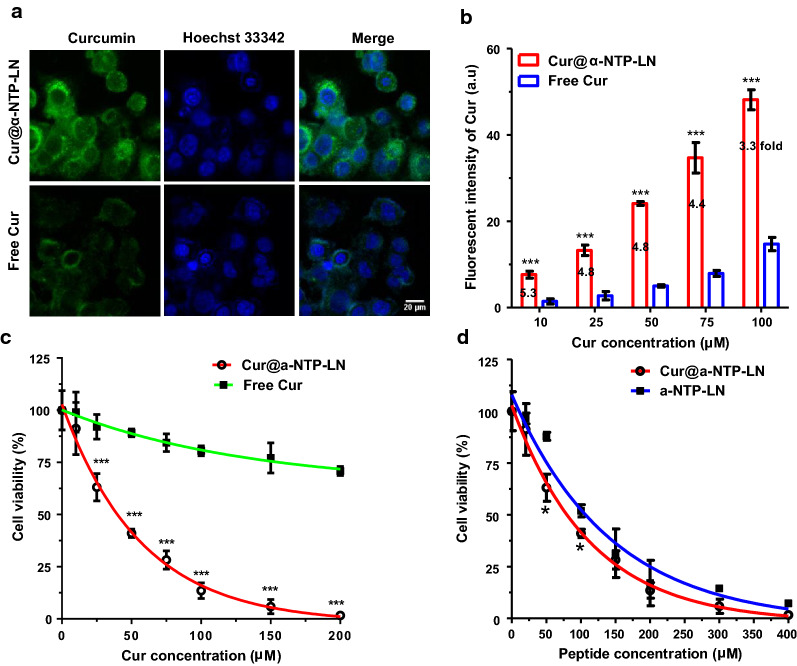
NPC targeting ability and cytotoxicity of Cur@α-NTP-LNs. **a** Confocal images of 5-8F cells incubated with 10 µM Cur@α-NTP-LNs and free Cur. Scale bar = 20 µm. **b** Quantitative flow cytometry analysis of the fluorescence intensity of Cur in 5-8F cells treated with a serial concentration of Cur@α-NTP-LNs and free Cur for 3 h (n = 4). **c**, **d** Proliferation assay evaluating the cytotoxic effect on 5-8F cells of a serial Cur concentration of **c** free Cur and Cur@α-NTP-LNs or a serial peptide concentration of **d** Cur@α-NTP-LNs and α-NTP-LNs for 24 h (n ≥ 3). Results are presented as mean ± s.d. ^***^p < 0.001 and ^*^p < 0.05

Confocal microscopy and flow cytometry were used to investigate the targeted delivery ability of Cur@α-NTP-LNs in releasing Cur to NPC. The binding affinity of Cur@α-NTP-LN and free Cur to human NPC 5-8F cells was evaluated by detecting the fluorescence of Cur. After 3 h of incubation with Cur@α-NTP-LNs, SR-B1^+^ 5-8F cells [25] displayed a strong Cur fluorescence signal mainly located in the cytoplasm (Fig. 2a). However, the fluorescence signal of free Cur in 5-8F cells was weak and located on the surface of the cell membrane, consistent with previous reports that small molecular Cur enters cells via passive diffusion [26]. Flow cytometry analysis further showed the higher uptake of Cur@α-NTP-LNs at each Cur concentration compared to free Cur (Fig. 2b). The uptake efficiency of Cur@α-NTP-LNs was increased in 5-8F cells in a concentration-dependent manner. Cur@α-NTP-LNs were more efficient in delivering Cur, with an approximate three–fivefold enhancement at various concentrations compared with free Cur (Fig. 2b). Together, the results indicated that the highly efficient delivery of Cur was due to the strong NPC-specific targeting of Cur@α-NTP-LN.

**Fig. 3:**
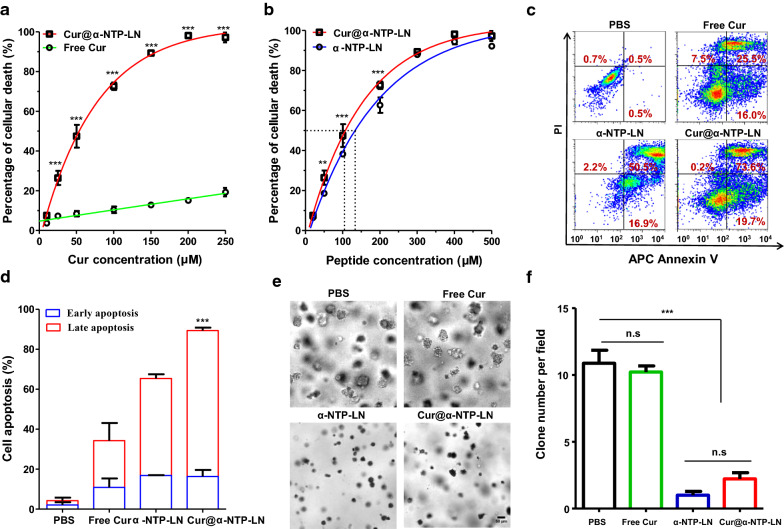
Cytotoxic effect and apoptosis induced by Cur@α-NTP-LNs. **a**, **b** Quantitative flow cytometric analysis of the mortality rates of 5-8F cells treated with a serial Cur concentration of **a** free Cur and Cur@α-NTP-LNs or a serial peptide concentration of **b** Cur@α-NTP-LNs and α-NTP-LNs (n ≥ 6). **c**, **d** Quantitative flow cytometry analysis of cell apoptosis rates of 5-8F cells treated with PBS or 100 µM free Cur, Cur@α-NTP-LNs, or peptide equivalent α-NTP-LNs (200 µM peptide) for 24 h, in which **c** is the dot plot presentation (APC-Annexin V/PI) of the representative data and **d** is the quantification of the apoptosis obtained in **c** after four independent experiments. Results are presented as mean ± s.d. (n = 4). ^***^p < 0.001 and ^**^p < 0.01. **e** Images of 5-8F cell colonies after the treatment with PBS or 25 µM free Cur, Cur@α-NTP-LNs, and peptide equivalent α-NTP-LNs (50 µM peptide). **f** Quantitative analyses of the colony number per field in each treatment group of **e**

The concentration-dependent cytotoxicity of Cur@α-NTP-LNs was further investigated on NPC cells. Cell proliferation assay showed that the proliferation rate of 5-8F cells was 41.0 ± 2.0% when treated with 50 μM (Cur concentration) Cur@α-NTP-LNs, while free Cur exhibited a significantly lower inhibitory effect on cell proliferation under the same conditions (n ≥ 3, *p* < 0.001, Fig. 2c). Furthermore, Cur@α-NTP-LNs showed a significantly higher inhibition rate on 5-8F cell proliferation at 50 and 100 μM of peptide concentration compared to that of α-NTP-LNs (n ≥ 3, *p* < 0.5, Fig. 2d). After 24 h incubation with nanoparticles, flow cytometry analysis also showed that the death rate of 5-8F cells was correlated with the concentration of the nanoparticles (Fig. 3a and b). Free Cur induced only an approximate 20% cell death even at a high concentration (250 µM), which was significantly lower than that induced by Cur@α-NTP-LNs in terms of cytotoxicity to 5-8F cells (n ≥ 8, *p* < 0.001, Fig. 3a). The half-maximum inhibitory concentration (IC_50_) of Cur@α-NTP-LNs and α-NTP-LNs associated to 5-8F cells was 104.1 ± 2.5 µM and 129.6 ± 3.7 µM, respectively (n ≥ 6, Fig. 3b). Cur@α-NTP-LNs exerted a significantly higher killing effect on 5-8F cells at 50, 100, and 200 μM of peptide concentration compared to the effect of α-NTP-LNs. The results confirmed that Cur@α-NTP-LNs exerted an enhanced inhibitory effect on 5-8F cells due to the potential synergistic interaction of α-NTP-LNs combined with the anti-cancer agent Cur.

Next, 5-8F cells were incubated with 100 µM free Cur or Cur@α-NTP-LNs (100 µM Cur, 200 µM peptide) or 200 µM of α-NTP-LNs for 24 h, stained with APC Annexin V and PI, and subjected to flow cytometry analysis to investigate the mechanism of Cur@α-NTP-LNs-induced cell death (Fig. 3c). The results showed that Cur@α-NTP-LNs induced cell death in 90.0 ± 2.6% of cells through apoptosis, with 16.3 ± 3.3% of cells in early apoptosis and 73.1 ± 1.5% of cells in late apoptosis (n = 4, Fig. 3d). α-NTP-LNs induced 65.0 ± 2.1% of apoptosis, which was consistent with our previous report [20], while only 34.3 ± 10.3% of apoptosis was found in response to free Cur. Together, these results indicated that the combination of α-NTP-LNs and Cur synergistically enhanced the apoptotic rate in 5-8F cells.

The anti-cancer activity of Cur@α-NTP-LNs was also evaluated through mobility inhibition and colony formation assay. The wound-healing assay revealed that 5-8F cells treated with Cur@α-NTP-LNs or peptide equivalent α-NTP-LNs significantly reduced cell motility compared to free Cur-treated cells (Cur equivalent) (Additional file 1: Fig. S3). As regard the colony formation assay, cells treated with PBS control resulted in the formation of large colonies, while 25 µM free Cur treatment resulted in less inhibitory effects on colony formation in 5-8F cells. However, Cur@α-NTP-LNs formulation exerted a more evident reduction in colony formation compared to the effect of free Cur (Fig. 3e, f). Altogether, these results further demonstrated that Cur@α-NTP-LNs exerted a high anti-cancer activity on 5-8F cells.

### Targeted Cur delivery of Cur@α-NTP-LNs in vivo

**Fig. 4:**
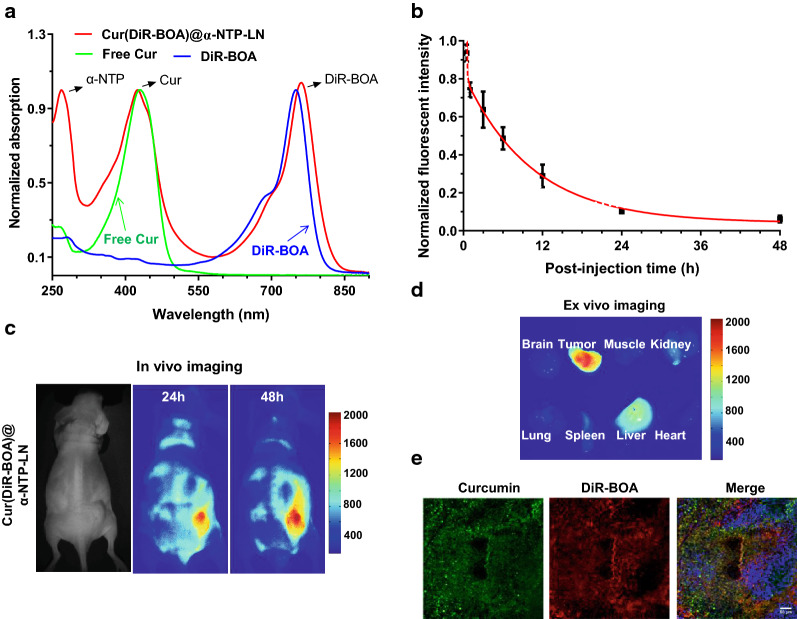
Evaluation of Cur targeted delivery of Cur@α-NTP-LNs in vivo*.* **a** UV–visible absorption spectrum of Cur(DiR-BOA)@α-NTP-LNs, free Cur, and free DiR-BOA in PBS solution at room temperature. **b** Blood circulation of Cur(DiR-BOA)@α-NTP-LNs in normal nude mice (n = 5). **c** Whole-body imaging of 5-8F tumor-bearing mice at 24 and 48 h after intravenous injection of Cur(DiR-BOA)@α-NTP-LNs. **d** ex vivo images of the tumor and normal organs (brain, muscle, kidney, lung, spleen, liver, and heart) after imaging. **e** Confocal imaging analysis of the co-localization of Cur (Green) and DiR-BOA (Red) in tumor sections pre-stained with DAPI (blue) before multispectral microscopy. Scale bar = 50 µm. Results are presented as mean ± s.d

The lipid-anchored near-infrared fluorophore DiR-BOA was incorporated into the core of Cur@α-NTP-LNs, named as Cur(DiR-BOA)@α-NTP-LNs, and the synthesized Cur(DiR-BOA)@α-NTP-LNs was tested for its UV–vis absorbance spectra to evaluate the NPC-targeting property of Cur@α-NTP-LNs in vivo (Fig. 4a). The fluorescence imaging of semi-native SDS-PAGE gels confirmed its in vitro stability (Additional file 1: Fig. S4). A pharmacokinetic study was conducted to estimate the circulation of Cur(DiR-BOA)@α-NTP-LNs in the blood. The pharmacokinetic profile, obtained by fitting the data to a two-compartment model, showed that the half-life (t_1/2α_) of Cur(DiR-BOA)@α-NTP-LNs during the initial elimination phase was 5.8 min, and its half-life (t_1/2β_) in the terminal elimination phase was 7.2 h (Fig. 4b). Thus, the loading of Cur into α-NTP-LNs did not affect its in vivo pharmacokinetics.

After intravenous injection of Cur(DiR-BOA)@α-NTP-LNs into 5-8F tumor-bearing mice, the fluorescence signals of DiR-BOA were monitored with a homemade whole-body fluorescence imaging system. Fluorescence signals at the tumor sites at 24 h post-injection were observed and gradually increased with time due to the selective accumulation of Cur(DiR-BOA)@α-NTP-LNs in the tumor regions (Fig. 4c). The ex vivo fluorescence evaluation of the dissected tumors and normal tissues (brain, muscle, kidney, lung, spleen, liver, and heart) showed strong NIR fluorescence signals in the tumor region in Cur(DiR-BOA)@α-NTP-LNs-treated group (Fig. 4d). Confocal imaging of the frozen tumor slices from the Cur(DiR-BOA)@α-NTP-LN-treated group displayed strong fluorescence signals of Cur and DiR-BOA, demonstrating that Cur@α-NTP-LNs penetrated the solid 5-8F tumors and efficiently delivered Cur into the tumor site (Fig. 4e).

### Cur@α-NTP-LNs inhibited the growth of 5-8F tumors in vivo

**Fig. 5:**
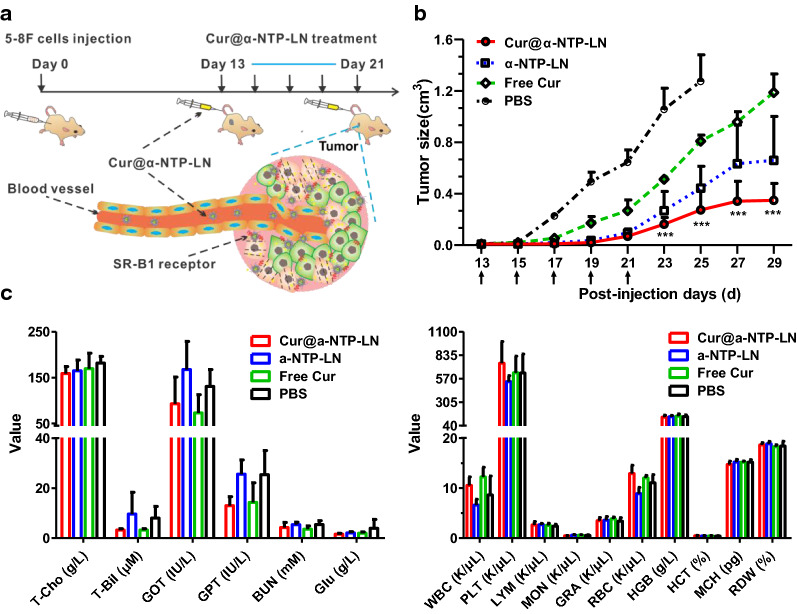
Cur@α-NTP-LNs inhibited 5-8F subcutaneous tumor growth. **a** Schematic diagram of Cur@α-NTP-LNs treatment on 5-8F subcutaneous tumors. **b** Tumor volume in each group over time. The tumor volume in the Cur@α-NTP-LNs-treated group was significantly smaller than that in other groups (n = 5). **c** Evaluation of the side effects of Cur@α-NTP-LNs, α-NTP-LNs, free Cur or PBS in vivo. (n ≥ 3). Results are presented as mean ± s.d. ***p < 0.001

5-8F subcutaneous tumor-bearing mice were prepared and randomly divided into four groups on day 13 from the injection of 5-8F tumor cells to evaluate the therapeutic efficacy of Cur@α-NTP-LNs on NPC tumors. Mice with a tumor volume of over 20 mm^3^ were intravenously injected with PBS or 125 nmol free Cur, Cur@α-NTP-LNs, or peptide equivalent α-NTP-LNs on day 13, and the intravenous injection was repeated every other day for 5 times (Fig. 5a). The tumor volume in the PBS-treated group on day 25 almost reached 1500 mm^3^ endpoint, and the mice were euthanized for humane reasons. The mean tumor volumes on day 29 were significantly different among Cur-treated group (1187 ± 145.8 mm^3^), α-NTP-LNs-treated group (660 ± 343 mm^3^), and Cur@α-NTP-LNs-treated group (348 ± 133 mm^3^) (n = 5, *p* < 0.001, Fig. 5b). The growth of 5-8F tumors was inhibited by Cur@α-NTP-LNs, with more than 71% and 47% inhibition relative to Cur- and α-NTP-LNs-treated control groups, respectively, suggesting a synergistically enhanced NPC-specific anti-cancer effects of Cur@α-NTP-LNs on tumor growth.

Blood was collected on days 25 and 29 from 5-8F cell injection for hemanalysis and biochemical analysis to evaluate the toxicological potential of Cur@α-NTP-LNs. Figure 5c shows no statistically significant differences in the hepatic and renal function [e.g. aspartate aminotransferase (GOT), alanine aminotransferase (GPT), glucose (Glu), urea nitrogen (BUN), total cholesterol (T-Cho), and total bilirubin (T-Bil)], and hemanalysis parameters [e.g. white blood cells (WBC), blood platelets (PLT), leukocytes (LYM), monocytes (MON), granulocytes (GRA), red blood cells (RBC), hemoglobin (HGB), hematocrit (HCT), and corpuscular hemoglobin (MCH), and red blood cell volume distribution width (RDW)] among the four treatment groups (5 mice per group). Thus, no noticeable side effects occurred after the systemic administration of Cur@α-NTP-LNs, providing a potential use of Cur@α-NTP-LNs as an effective and safe therapeutic agent for the therapy to cure NPC.

### Cur@α-NTP-LNs delayed NPC lung metastasis

**Fig. 6:**
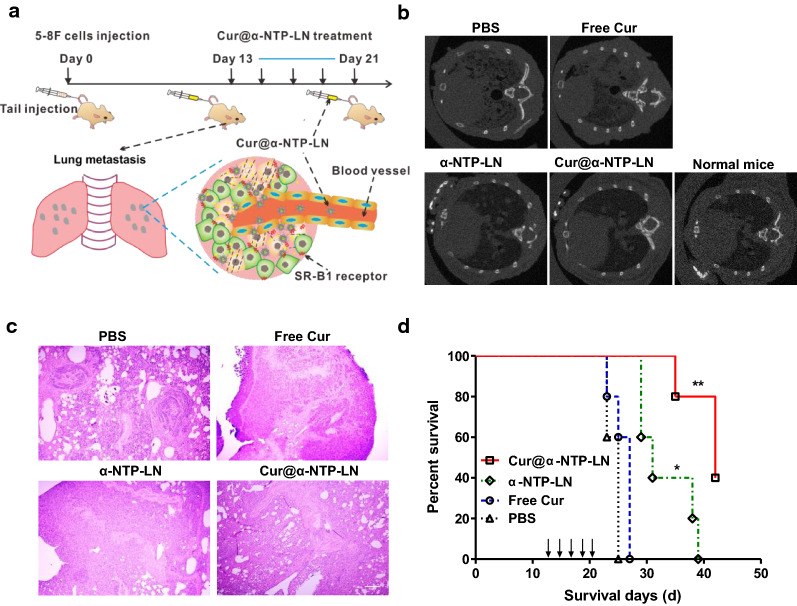
Cur@α-NTP-LNs delayed the progression of NPC lung metastasis. **a** Schematic diagram of Cur@α-NTP-LNs treatment on NPC lung metastasis. **b** X-ray computed tomography scan for the detection of pulmonary metastases in normal mice and mice after treatment with PBS or 125 nmol of free Cur, Cur equivalent Cur@α-NTP-LNs or peptide equivalent α-NTP-LNs at day 25 after 5-8F cell injection. **c** Histopathological analyses of H&E-stained lung tissues from mice treated with Cur@α-NTP-LNs, α-NTP-LNs, Cur or PBS at day 25 after 5-8F cell injection. Scale bar = 50 µm. **d** Survival curve of mice with NPC lung metastases after treatment with PBS or 125 nmol free Cur, Cur equivalent Cur@α-NTP-LNs or peptide equivalent α-NTP-LNs (n = 5)

5-8F cells were intravenously injected into mice to detect the ability of Cur@α-NTP-LNs to inhibit pulmonary metastases, and the mice were randomly divided into 4 groups and treated with an intravenous injection of PBS or 125 nmol free Cur, Cur@α-NTP-LNs or peptide equivalent α-NTP-LNs through the tail vein. The treatments were performed on day 13, 15, 17, 19, and 21 after 5-8F cell injection (Fig. 6a). Three mice in free Cur, Cur@α-NTP-LNs- and α-NTP-LNs-treated groups were separately used to perform an X-ray computed tomography (CT) scan on day 25. Axial CT scan results revealed the presence of large and numerous lung metastases in PBS- and free Cur-treated mice, small pulmonary nodules of various sizes in α-NTP-LNs-treated mice, and no significant difference with the lung of normal mice in the Cur@α-NTP-LNs-treated mice (Fig. 6b). The mice were sacrificed after the confirmation of the presence of metastases by CT, and sections of lung tissues were stained with H&E to evaluate lung metastasis. The H&E images showed apparent primary malignancies in PBS control mice, plenty of macroscopic malignant nodules in Cur-treated mice, and few macroscopic malignant nodules in α-NTP-LNs-treated mice (Fig. 6c), indicating that Cur@α-NTP-LNs efficiently inhibited NPC lung metastasis.

Next, an NPC lung metastasis model was prepared to further investigate the therapeutic efficacy of Cur@α-NTP-LNs in the treatment of NPC lung metastasis by the injection of mRFP-5-8F cells on the tail vein of athymic nude mice (nu/nu). An amount of 125 nmol free Cur, Cur@α-NTP-LNs or peptide equivalent α-NTP-LNs were injected at day 13 after the injection of mRFP-5-8F cells, and the treatment experiments were started on days 13, 15, 17, 19, and 21. All mice were sacrificed on day 35, and fluorescent imaging was performed. The whole-body fluorescence imaging revealed that lung metastases only occurred in the Cur-treated group (Additional file 1: Fig. S5a). Lung tissues in all treated mice were exposed and imaged under red fluorescence illumination. Red fluorescence images showed that small lung metastases were easily visible and detected in the Cur-treated group, whereas fluorescence signals in the Cur@α-NTP-LNs- and α-NTP-LNs-treated group were almost negligible (Additional file 1: Fig. S5b). H&E staining of lung tissues was performed to verify the inhibitory effect of Cur@α-NTP-LNs and α-NTP-LNs on lung metastasis. The necropsy results showed plenty of malignant nodules in Cur-treated mice and few macroscopic malignant nodules in the α-NTP-LNs-treated mice, while no malignant nodules were detected in Cur@α-NTP-LNs-treated mice (Additional file 1: Fig. S5c). These results showed that Cur@α-NTP-LNs exerted synergistic enhanced inhibitory effect on NPC lung metastasis compared to free Cur and α-NTP-LNs.

5-8F lung metastatic mice models were prepared and monitored on day 27 after 5-8F cell injection and different treatments to evaluate the effect of Cur@α-NTP-LNs on survival rates. All the PBS-treated control mice (n = 5) and free Cur-treated mice (n = 5) died, whereas α-NTP-LNs- and Cur@α-NTP-LNs-treated mice were alive (n = 5, *p* < 0.05, Fig. 6d). In addition, 60% of the Cur@α-NTP-LNs-treated mice were still alive at day 42 after cell injection, and all the other treated mice were dead (n = 5, *p* < 0.01, Fig. 6d). These results revealed that Cur@α-NTP-LNs significantly delayed the development of lung metastases from NPC cells and extended the survival rate of the mice compared to α-NTP-LNs and free Cur.

## Discussion

Cur is a phytochemical compound extracted from the rhizome of turmeric, with a remarkable therapeutic potential in the treatment of NPC [27, 28]. Extensive preclinical and clinical studies in the past decades have indicated the therapeutic effects of Cur in curing cancer [15, 29]. Some proof-of-concept studies demonstrated that Cur efficiently sensitizes NPC cells to chemotherapeutic agents and radiation therapy [28, 30]. Research studies proved that Cur not only suppresses cell proliferation, metastasis, and tumor angiogenesis, but also induces cell apoptosis through the modulation of different molecular targets [31, 32]. However, fast systemic elimination and low bioavailability hinder its clinical application [33]. The incorporation of Cur into nanoparticles can enhance its bioavailability at the tumor sites through a passive or active mechanism, being beneficial for patients [15]. This study focused on the development of a targeted Cur nanoparticle formulation to treat advanced NPC.

Cur was encapsulated into α-NTP-LNs to form a new sub-30 nm NPC-targeting therapeutic lipid nanoparticle Cur@α-NTP-LNs, which combined the NPC-specific therapeutic effects of α-NTP-LNs with the cancer chemopreventive effects of Cur. α-NTP-LNs exhibit SR-B1 targeting, abundantly expressed in 5-8F cells, and exerts cytotoxicity through the induction of apoptosis and autophagy [20]. However, flow cytometry analysis showed that Cur@α-NTP-LNs not only keep the ability to target SR-B1 but also exerted a synergistically enhanced suppression of 5-8F cell proliferation, motility, and colony formation compared to free Cur and α-NTP-LNs (Fig. 3). Furthermore, Cur@α-NTP-LNs achieved over 71% and 47% inhibitory effect on 5-8F tumor growth when compared to free Cur and α-NTP-LNs respectively, confirming that Cur@α-NTP-LNs with its combined anti-cancer activity of α-NTP-LNs and Cur synergistically inhibited NPC tumor growth (Fig. 5). The substantially improved therapeutic effects of Cur@α-NTP-LNs in vivo might be attributable to the synergistic inhibition of tumor cell growth and tumor angiogenesis because the higher inhibitory effect of Cur@α-NTP-LNs than that of α-NTP-LNs on tumor growth was observed on day 23 after 5-8F cells implantation (Fig. 5). Cur@α-NTP-LNs used in the 5-8F lung metastasis models showed significantly improved survival rates compared to those of α-NTP-LNs, which further confirmed its synergistically enhanced suppression on tumor growth and angiogenesis (Fig. 6). Additionally, Cur@α-NTP-LNs treatment did not cause any detectable damage to liver and kidney function and cell membrane of RBCs, as revealed by hemanalysis and biochemical analysis (Fig. 5). These properties might encourage the further development of Cur@α-NTP-LNs formulations for the preclinical application of NPC-specific targeted delivery.

Free Cur could be loaded into the hydrophobic core of α-NTP-LNs thanks to the lipophilic property of curcumin, which not only increased its aqueous solubility and stability, but also protected its bioactivity during the in vivo delivery of cancer therapeutics. The spontaneous oxidative degradation of Cur is also a primary factor contributing to its low bioavailability[34]. Curcumin undergoes autoxidation very easily, inactivated by peroxidases and oxidizing agents, and degradation at physiological conditions [35]. Cur exerts its anti-cancer effect through oxidative degradation to modulate reactive oxygen species (ROS) generation [36]. Most conventional nanoparticles lack ROS scavenging properties and cannot protect Cur from ROS-mediated oxidative degradation [15, 37]. α-NTP-LNs were composed of amphipathic α-helical peptides, and the amphipathic apoA-I mimetic peptide exerts anti-cancer effect as a direct scavenger of ROS [38, 39]. Thus, Cur@α-NTP-LNs might protect the encapsulated Cur from oxidative degradation, improve its poor bioavailability and increase its tumor-targeting ability via targeted delivery.

Various Cur nanoformulations have been developed based on dispersed or precipitation processes for the accurate cancer imaging and effective treatment [40]. Although these nanoparticles confer many advantages over that of free chemotherapeutic agents, the lack of tissue specificity is still the main drawback of chemotherapy because most reported Cur nanoparticles can only passively accumulate in the tumor site via enhanced permeability and retention (EPR) effect [36]. Nanoparticles presenting targeting moieties (e.g. antibodies, peptides, or nucleic acid/aptamers) on the surface can achieve high targeting specificity, while avoiding non-specificity binding [36, 41]. Murali et al. conjugated PSMA whole antibody to PLGA-CUR nanoparticle for the targeted delivery of Cur to prostate cancer [42]. However, the batch-to-batch reproducibility and in vivo toxicity of conventional Cur nanoparticles may curb their clinical translation. Our Cur@α-NTP-LNs nanoformulation was generated with a simple and robust approach and did not exert detectable toxicity in vivo, being potentially promising in the treatment of NPC and its distant metastases in clinical practice. Furthermore, the ultra-small biocompatible nanoparticles loading tumor antigen peptides revealed an enhancement of antigen presentation by dendritic cells through the SR-B1 pathway [43–45]. Future studies with combined delivery of chemotherapeutic agents with imaging contrast agents, tumor-specific antigenic peptides or antibody fragments in a single α-NTP-LNs nanoparticle could reveal the potentially more effective way of treating NPC.

## Conclusions

In conclusion, a sub-30 nm biocompatible lipid nanoparticle Cur@α-NTP-LNs was constructed, exhibiting SR-B1 targeting ability and synergistically enhanced inhibitory effect on NPC tumor growth and its metastasis. The newly formed nanoparticles Cur@α-NTP-LNs successfully encapsulated Cur to increase its solubility, and its specifically deliver into the center of solid NPC tumors, with enhanced anti-cancer activities due to its ultra-small size. Furthermore, our results showed that Cur@α-NTP-LNs were not only effective in targeting NPC cells, suppressing cellular proliferation, and inducing cell apoptosis in vitro, but also exhibited a remarkable inhibitory effect on NPC tumors and their metastasis in vivo. Thus, the overall experimental evidence suggested that Cur@α-NTP-LNs formulation might be an excellent promise for the preclinical treatment of NPC tumors due to its low toxicity and prominent therapeutic effects.

## Supplementary Information

**Additional file 1**: **Figure S1**. Particle size distribution of Cur@α-NTP-LNs. Dynamic light scattering (DLS) measurement showing the intensity weighted particle size of Cur@α-NTP-LNs that varied from 15.2 to 119.8 nm with the increasing amount of α-NTP peptide. **Figure S2**. Excitation and emission spectra of free curcumin in PBS solution at room temperature. **Figure S3**. Inhibition of the proliferation of 5-8F cells treated with PBS, 50 µM free Cur, Cur equivalent Cur@α-NTP-LNs, or peptide equivalent α-NTP-LNs (100 µM) at 24 h. Scale bar = 100 µm. **Figure S4**. Semi-native SDS-PAGE assay and fluorescence imaging to evaluate the stability of Cur(DiR-BOA)@α-NTP-LNs. **Figure S5**. Anti-cancer effects of Cur@α-NTP-LNs on mRFP-5-8F lung metastasis. (a) Overlay of bright field and color fluorescence images showing mRFP-5-8F tumor metastasis that occurred in free Cur-treated mice but undetectable in both Cur@α-NTP-LNs- and α-NTP-LNs-treated mice by necropsy. (b) Fluorescence imaging of lung tissues collected from mice in a. (c) Histopathological analyses of H&E-stained lung tissues from mice treated with 135 nmol free Cur, Cur equivalent Cur@α-NTP-LNs or peptide equivalent α-NTP-LNs at day 35 after mRFP-5-8F cell injection. Scale bar = 30 µm.
