# Supplementary material for: Curcumin loaded sub-30 nm targeting therapeutic lipid nanoparticles for synergistically blocking nasopharyngeal cancer growth and metastasis
Source: J Nanobiotechnology. 2021 Jul 28;19:224. doi: 10.1186/s12951-021-00966-6 (PMC8317404; doi:10.1186/s12951-021-00966-6)
Supplement: Supplementary file 1 — Additional file 1: Figure S1. Particle size distribution of Cur@α-NTP-LNs. Dynamic light scattering (DLS) measurement showing the intensity weighted particle size of Cur@α-NTP-LNs that varied from 15.2 to 119.8 nm with the increasing amount of α-NTP peptide. Figure S2. Excitation and emission spectra of free curcumin in PBS solution at room temperature. Figure S3. Inhibition of the proliferation of 5-8F cells treated with PBS, 50 µM free Cur, Cur equivalent Cur@α-NTP-LNs, or peptide equivalent α-NTP-LNs (100 µM) at 24 h. Scale bar = 100 µm. Figure S4. Semi-native SDS-PAGE assay and fluorescence imaging to evaluate the stability of Cur(DiR-BOA)@α-NTP-LNs. Figure S5. Anti-cancer effects of Cur@α-NTP-LNs on mRFP-5-8F lung metastasis. (a) Overlay of bright field and color fluorescence images showing mRFP-5-8F tumor metastasis that occurred in free Cur-treated mice but undetectable in both Cur@α-NTP-LNs- and α-NTP-LNs-treated mice by necropsy. (b) Fluorescence imaging of lung tissues collected from mice in a. (c) Histopathological analyses of H&E-stained lung tissues from mice treated with 135 nmol free Cur, Cur equivalent Cur@α-NTP-LNs or peptide equivalent α-NTP-LNs at day 35 after mRFP-5-8F cell injection. Scale bar = 30 µm. [file 12951_2021_966_MOESM1_ESM.docx]

**Supplementary Figures**


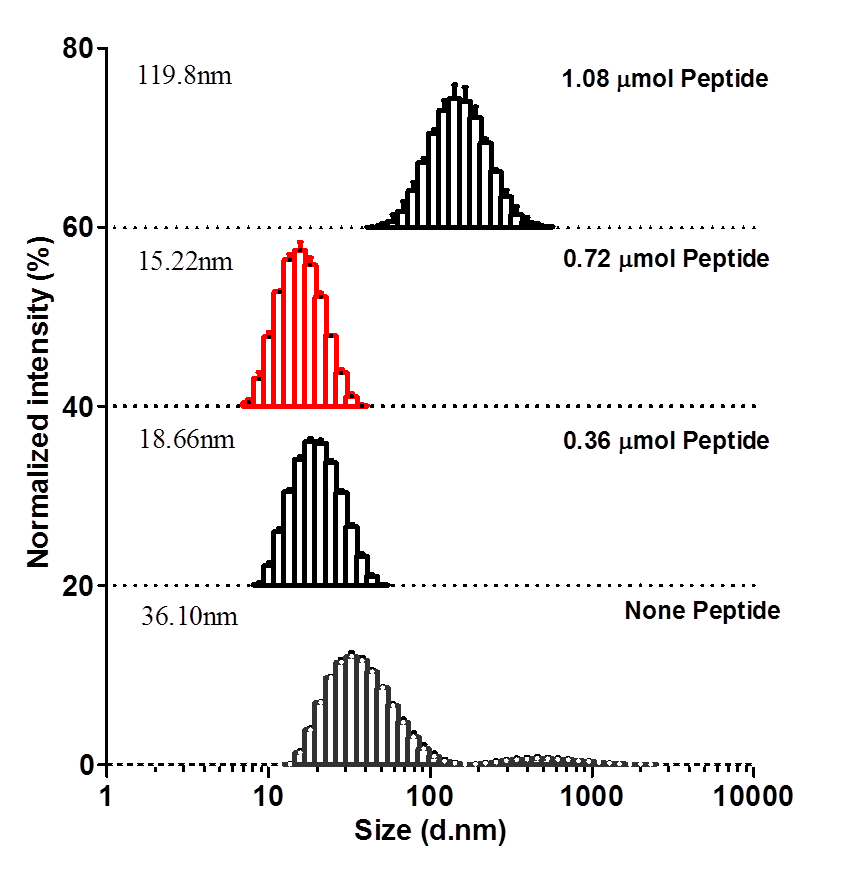


**Figure S1. Particle size distribution of Cur@α-NTP-LNs**. Dynamic light scattering (DLS) measurement showing the intensity weighted particle size of Cur@α-NTP-LNs that varied from 15.2 to 119.8 nm with the increasing amount of α-NTP peptide.


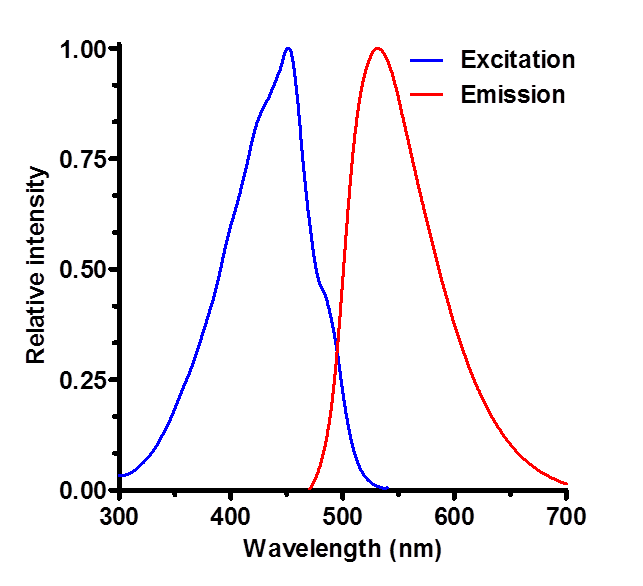


**Figure S2.** Excitation and emission spectra of free curcumin in PBS solution at room temperature.

**Figure S3.** Inhibition of the proliferation of 5-8F cells treated with PBS, 50 µM free Cur, Cur equivalent Cur@α-NTP-LNs, or peptide equivalent α-NTP-LNs (100 µM) at 24 h. Scale bar = 100 µm.

**Figure S4.** Semi-native SDS-PAGE assay and fluorescence imaging to evaluate the stability of Cur(DiR-BOA)@α-NTP-LNs.

**Figure S5.** Anti-cancer effects of Cur@α-NTP-LNs on mRFP-5-8F lung metastasis. (a) Overlay of bright field and color fluorescence images showing mRFP-5-8F tumor metastasis that occurred in free Cur-treated mice but undetectable in both Cur@α-NTP-LNs- and α-NTP-LNs-treated mice by necropsy. (b) Fluorescence imaging of lung tissues collected from mice in a. (c) Histopathological analyses of H&E-stained lung tissues from mice treated with 135 nmol free Cur, Cur equivalent Cur@α-NTP-LNs or peptide equivalent α-NTP-LNs at day 35 after mRFP-5-8F cell injection. Scale bar = 30 µm.
